# Strangulated Hernia in Man of 85—Operation

**Published:** 1905-03

**Authors:** Geo. Harwood

**Affiliations:** Johnson City, Texas


For Texas Medical Journal.

### Strangulated Hernia In Man of 85—Operation.

BY GEO. HARWOOD, M. D., JOHNSON CITY, TEXAS.

The following record of case I send to you as being rare, considering the age of patient and length of time before relief was obtained:

Was called to Mr. S., who lived fifteen miles from my office, on the night of November 5th, getting there about 12 midnight. I found an old gentleman in his 85th year suffering from strangulated hernia. On asking him when he discovered that he could not return his rupture, he told me that it was the evening before, and that he had been trying most of that night and next day to return it and had made it very tender; had also taken several pills and doses of salts.

I failed to return the bowel, using every means of relaxation and gentle taxis, and told him that I would be obliged to operate. He begged for twelve hours longer, which I gave him, having to return home for instruments and anesthesia.

On operating, I found the bowel to be greatly congested, almost black. I returned it, washed out the sac, and finished the operation in as short time as possible, as he was not taking the anesthetic very well, and under such antiseptic precautions as were possible, in spite of which part of the scrotum sloughed away.

The old gentleman made a good recovery, and is now following his usual occupation of school teacher, and occasionally takes a ride horseback.

